# Confirmation of *Rickettsia conorii* Subspecies *indica* Infection by Next-Generation Sequencing, Shandong, China

**DOI:** 10.3201/eid2710.204764

**Published:** 2021-10

**Authors:** Nannan Xu, Wei Gai, Yan Zhang, Wei Wang, Gang Wang, Gregory A. Dasch, Marina E. Eremeeva

**Affiliations:** Qilu Hospital of Shandong University, Jinan, Shandong, China (N. Xu, G. Wang);; Gene Research Institute, WillingMed Technology (Beijing) Co. Ltd., Beijing, China (W. Gai, Y. Zhang);; The University of Texas MD Anderson Cancer Center, Houston, Texas, USA (W. Wang);; Centers for Disease Control and Prevention, Atlanta, Georgia, USA (G.A. Dasch);; Georgia Southern University, Statesboro, Georgia, USA (M.E. Eremeeva)

**Keywords:** *Rickettsia conorii*, *Rickettsia conorii* Subspecies *indica*, next-generation sequencing, vector-borne infections, rickettsia, Shandong Province, China, bacteria

## Abstract

We describe 3 similar cases of rickettsial disease that occurred after tick bites in a mountainous rural area of Shandong Province, China. Next-generation sequencing indicated the etiologic agent of 1 patient was *Rickettsia conorii* subspecies *indica*. This agent may be more widely distributed across China than previously thought.

Shandong is an eastern coastal province of China. Four natural-focal diseases—severe fever with thrombocytopenia syndrome, human granulocytic anaplasmosis, endemic typhus, and scrub typhus—are thought to have the most severe effects on human health in Shandong Province (*1*). However, as in other parts of China, exposure to rickettsial pathogens in eastern provinces is expected because of the prevalence of human-biting ticks (*2*,*3*). Specifically, Japanese spotted fever caused by *Rickettsia japonica* is endemic to Shandong; *R. japonica* and 2 other novel *Rickettsia* spp. were found in the Asian longhorned tick (*Haemaphysalis longicornis*) (*2*). Because rickettsioses have similar clinical manifestations but vary in severity (i.e., incidence of illness and death), laboratory investigation is essential for understanding the epidemiology of tick-borne diseases. We obtained sequences of *Rickettsia conorii* subspecies *indica* (ITTR) infection from 1 case; 2 other cases of spotted fever rickettsiosis (SFGR) with similar epidemiologic history and clinical features were treated at the same hospital (Appendix Table 1, Figure 1). This study was approved by the ethics committee of Qilu Hospital, of Shandong University, Jinan, Shandong, China. All patients signed consent forms.

## The Study

**Figure 1:**
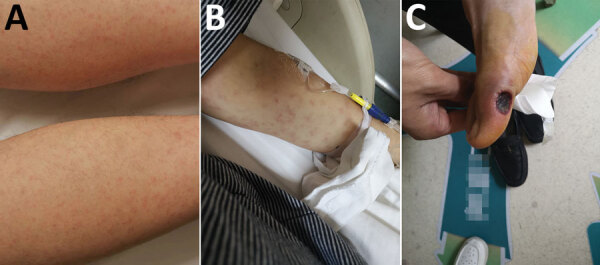
Skin manifestations of patients in study of confirmation of *Rickettsia conorii* subspecies *indica* infection by next-generation sequencing, Shandong, China. A) Rash in patient 1; B) rash in patient 2; C) eschar in patient 3

In the summer of 2019, a 53-year-old man (patient 1) was hospitalized with a 5-day history of fever (41°C), influenza-like symptoms, and generalized maculopapular rash (Figure 1, panel A). A farmer working in a rural mountainous area of Zibo, Shandong Province, he was bitten by a tick 6 days before onset of illness. At admission, clinical blood tests revealed elevated leukocyte count (12.91 × 10^9^ cells/L) with neutrophilia (90.5%) and thrombocytopenia (73 × 10^9^/L), as well as increased procalcitonin (3.870 ng/mL) and C-reactive protein (38.31 mg/mL). Rickettsiosis was suspected, and oral minocycline was prescribed on the second day after admission. Symptoms subsided after 2 days of treatment; the patient was discharged from the hospital 6 days later. Serum samples collected on days 8 and 24 after onset of illness tested positive for *Rickettsia conorii* IgG (titers 1,024 at day 8 and 16,384 at day 24) by immunofluorescence assay (IFA) (Fuller Laboratories, http://www.fullerlaboratories.com).

Patient 2, a 41-year-old female agriculture worker from Jinan, the capital of Shandong Province, came from an environment similar to that of patient 1. Patient 2 was hospitalized 18 days after a tick bite; symptoms were an 8-day history of fever (39°C), meningitis, and a sparsely spread purpuric rash (Figure 1, panel B). Intravenous doxycycline treatment was initiated 1 day after admission. Four days after admission, despite 2 days of treatment, the patient experienced seizures, coma, and cardiac arrhythmia. After 2 more days of intravenous doxycycline treatment, the patient improved and was discharged 4 days later. Serum samples collected on days 9 and 22 after onset of illness tested positive for *R. conorii* IgG by IFA (titers 128 at day 9 and 1,024 at day 22).

Patient 3, a 45-year-old woman, had a history of travel to a farming area in Tai’an, Shandong Province, and was bitten by a tick 8 days before onset of illness. At admission, she had a 5-day history of fever (39°C). She did not have rash but had an ulcerated eschar on her right foot (Figure 1, panel C). Blood tests at hospital admission revealed elevated leukocyte count (10.12 × 10^9^ cells/L), procalcitonin (0.108 ng/mL), and C-reactive protein (46.39 mg/mL). The patient was treated with minocycline beginning the next day after admission; she began to improve on day 3 of treatment and was discharged after 3 more days. Serum samples collected on days 9 and 20 after onset of illness tested positive for *R. conorii* IgG by IFA (titers 64 at day 9 and 1,024 at day 20).

Conventional bacterial cultures of blood samples collected at admission yielded negative results for all 3 patients, as did viral nucleic acid detection of pharyngeal swab samples. Results of serologic ELISA tests for *Coxiella burnetii* phase II IgG (IBL International GmbH, https://www.ibl-international.com), *Rickettsia typhi* IgM (Fuller Laboratories), and *Orientia tsutsugamushi* IgM (InBios International, Inc., https://inbios.com) were all negative.

**Figure 2:**
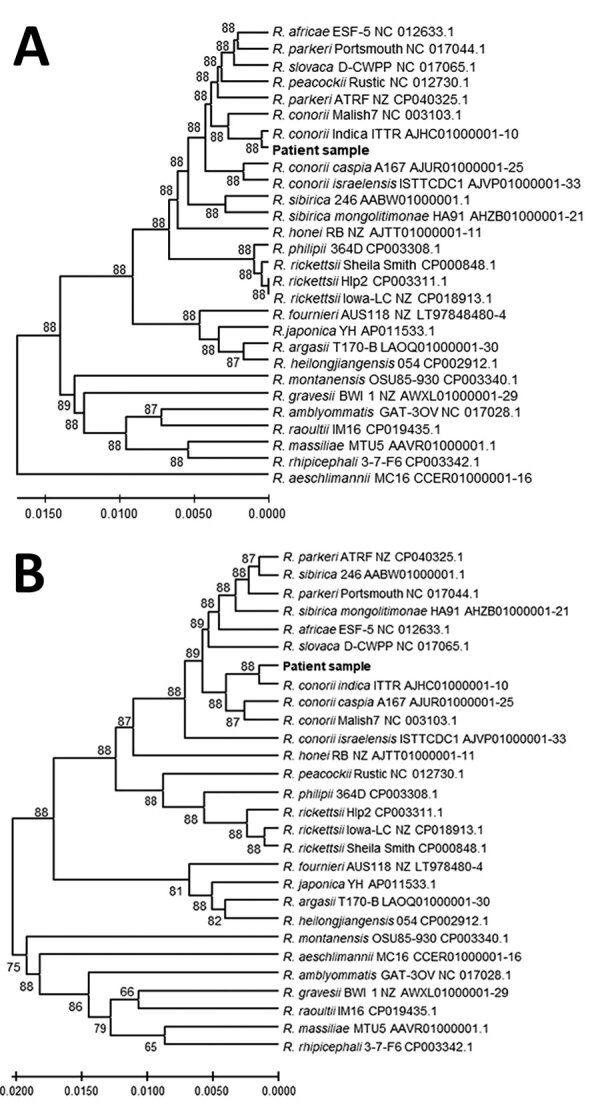
Genetic relationships of the spotted fever group rickettsia detected in blood of patient 1 in study of confirmation of *Rickettsia conorii* subspecies *indica* infection by next-generation sequencing, Shandong, China. This analysis used concatenated sequences from 27 spotted fever rickettsial genomes homologous to the patient sequences (shown in bold text). A) Analysis of 1,379 positions in the tRNA-associated sequences; B) analysis of 1,519 positions in the protein gene–associated sequences. Each tree was constructed upon concatenation of 6 different genome sites (Appendix Table 2); the consensus of reads from sites with overlapping reads was used. The evolutionary relationships were inferred by using UPGMA implemented in MEGA X (*15*). The optimal trees are shown. The percentage of replicate trees in which the taxa clustered together in the bootstrap test (500 replicates) are shown next to the branches. The evolutionary distances computed by using the Kimura 2-parameter method are in the units of the number of base substitutions per site. The proportion of sites where >1 unambiguous base is present in >1 sequence for each descendent clade is shown next each internal node in the tree. All ambiguous positions were removed for each sequence pair (pairwise deletion option). Scale bars indicate the percentage of nucleotide variation between the sequences.

To identify the potential causative pathogen, we performed next-generation sequencing (NGS) on the Ion Torrent platform (Thermo Fisher Scientific, https://www.thermofisher.com) by using DNA extracted from the peripheral blood of patient 1, collected on day 7 after onset of fever and before administration of antimicrobial drugs. The sequencing data are deposited at the National Center for Biotechnology Information Sequence Read Archive (accession no. SRR10855057). We mapped those sequences to *R. conorii* ITTR (Appendix Figure 2). Coverage was low except for 16S and 23S rRNA genes, but matching sequences were found across the ITTR genome and to other *Rickettsia* genomes (data not shown). We identified reads mapping to specific *Rickettsia* genomes by using BLAST (https://blast.ncbi.nlm.nih.gov/Blast.cgi) (Appendix Table 2). We identified the *Rickettsia*-specific 16S rRNA gene sequences with the Ribosomal Database Project Classifier by using Geneious Prime 19 (Geneious, https://www.geneious.com) (Appendix Table 2). Moreover, we identified sequence reads matching 3 genes commonly used for speciation of *Rickettsia* (*glt*A, *omp*A, *omp*B); 6 other proteins; and 1 pseudogene, *rnp*B, and containing or flanking 20 of the 33 rickettsial tRNAs (33 reads) (Figure 2; Appendix Table 2). Many sequence reads mapped most closely to ITTR or to ITTR and its closest relative, *R. conorii conorii* Malish 7; sequence reads mapped less frequently to the other subspecies, *R. conorii caspia* and *R. conorii israelensis.*

## Conclusions

Many tickborne rickettsiae have been described from China, including *R. heilongjiangiensis*, *R. sibirica* BJ-90, *R. sibirica mongolotimonae*, *R. monacensis*, *R. raoultii*, *R. slovaca*, *R. japonica*, *Candidatus* R. tarasevichiae, and other *Rickettsia* spp. of unknown pathogenicity (*2*,*4*). We molecularly confirmed a case of SFGR disease in eastern China caused by *R. conorii* subsp. *indica*. We identified 2 other serologically confirmed cases of SFGR with similar history of tick bite, similar clinical manifestations, and shared epidemiologic features.

NGS technology provided the specific etiology of SFGR in 1 of these patients. The single NGS read length exceeded the size of tRNAs, so they were informative for identification, but diagnostic sites were also obtained for protein fragments (Appendix Table 2). The sensitivity of NGS depends on the type of the clinical sample, the timing of collection, and desirability for depleting human DNA to improve sensitivity of pathogen detection by increasing the number of agent sequences (*5*).

*R. conorii* is divided taxonomically into 4 subspecies: *R. conorii conorii*, *R. conorii caspia*, *R. conorii israelensis*, and *R. conorii indica* (*6*). The members of this group exhibit substantial genome sequence similarity and shared antigenic makeup; however, the diseases they cause might be distinguished by specific clinical manifestations, rates of illness or death, and the areas of their endemicity and predominant tick vectors (*6*). PCR-confirmed clinical cases caused by ITTR have been diagnosed in India (*7*), Sicily (*8*) and Xinjiang Uygur Autonomous Region, China (GenBank accession nos. MG190327–9). Well-documented entomologic surveys indicate a broader area of circulation of this etiologic agent, extending beyond India and Pakistan (*9*) to Laos (*10*) and western provinces of China (*11*,*12*). In those areas, ITTR is associated either with *Rhipicephalus turanicus* (sheep tick) or *Rh. sanguineus* (brown dog tick) collected from pet dogs (*12*,*13*), suggesting a high probability of human exposure, given the proximity of these animals to human habitats. Our findings indicate that circulation of ITTR in Shandong Province and transmission to humans occurs in rural mountainous areas where the presence of both tick species has been documented (*3*,*14*). These findings suggest transmission of 1 or several SFGRs to humans might occur across China, thus requiring additional diagnostic and surveillance efforts that could lead to improved identification and management of patients with these infections.

Appendix Additional information about confirmation of *Rickettsia conorii* subspecies *indica* infection by next-generation sequencing, Shandong, China
