# Supplementary material for: Confirmation of Rickettsia conorii Subspecies indica Infection by Next-Generation Sequencing, Shandong, China
Source: Emerg Infect Dis. 2021 Oct;27(10):2691–4. doi: 10.3201/eid2710.204764 (PMC8462315; doi:10.3201/eid2710.204764)
Supplement: Appendix — Additional information about confirmation of Rickettsia conorii subspecies indica infection by next-generation sequencing, Shandong, China [file 20-4764-Techapp-s1.pdf]

Article DOI: https://doi.org/10.3201/eid2710.204764

Confirmation of *Rickettsia conorii* Subspecies *indica* Infection by Next-Generation Sequencing, Shandong, China

Appendix

Appendix Table 1. Clinical features and laboratory findings in 3 spotted fever patients, Shandong Province, China*

Characteristic, symptom, or laboratory parameter	Reference range	Patient 1	Patient 2	Patient 3
Demographics and exposure history				
Age, y		53	41	45
Sex		M	F	F
History of tick exposure		Yes	Yes	Yes
Time between tick bite and illness onset, d		6	10	8
Time to admission after disease onset, d		5	8	5
Clinical symptoms				
Elevated temperature		Yes (41°C)	Yes (39°C)	Yes (39°C)
Rash		Yes	Yes	No
Headache		Yes	Yes	Yes
Chills		Yes	Yes	Yes
Myalgia		Yes	Yes	Yes
Eschar		No	No	Yes
Nausea		No	Yes	Yes
Vomiting		No	Yes	Yes
Diarrhea		Yes	No	No
Neck stiffness		No	Yes	No
Duration of hospitalization, days		9	10	7
Time to follow-up visit, days		21	23	20
Hematologic test				
Leukocyte count, 10^9/L	3.5–9.5	12.91	5.48	10.12
Neutrophils, %	40–75	90.50	77.20	82.4
Eosinophils, %	0.4–8.0	0.00	0.20	0.40
Hemoglobin, g/L	130–175	151.0	86.0	136
Platelet count, 10^9/L	125–350	73	256	325
Procalcitonin, ng/mL	<0.1	3.870	0.064	0.108
C-reactive protein, mg/L	0–10	38.31	21.0	46.39
Erythrocyte sedimentation rate, mm/h	0–20	29.00	77.0	87
Biochemical test				
Alanine aminotransferase, U/L	21–72	66	19	75
Aspartate aminotransferase, U/L	17–59	62	23	65
Albumin, g/L	35–50	31	41	45
Total bilirubin, µmol/L	3–22	49	16	18
Conjugated bilirubin, µmol/L	0–5	7	0	4
Unconjugated bilirubin, µmol/L	0–19	21	14	18
Sodium, mmol/L	137–145	130	133	138
Calcium, mmol/L	2.1–2.55	2.03	2.23	2.43
Phosphorus, mmol/L	0.81–1.45	0.52	1.16	0.85
Urinalysis				
Erythrocytes (per high-power field)	0–10	329.56	35.20	NA
Blood	–	3+	3+	NA
Bilirubin	–	1+	–	NA.
Protein	–	1+	–	NA
Urobilinogen	–	3+	–	NA
Fecal analysis				
Blood	–	–	–	NA
Cerebrospinal fluid measurements				

Characteristic, symptom, or laboratory parameter	Reference range	Patient 1	Patient 2	Patient 3
Leukocyte count, per mm^3	$\leq$5	NA	1100	NA
Neutrophils, %		NA	76	NA
Lymphocytes, %		NA	18	NA
Glucose, mmol/L	2.5–4.5	NA	2.17	NA
Chlorine, mmol/L	120–130	NA	109	NA
Protein, g/L	0.15–0.45	NA	6.71	NA

*NA, not available (not performed or not reported); +, positive; –, negative.

Appendix Table 2. Characteristics of Ion Torrent next-generation sequencing reads mapping with high similarity to the *Rickettsia* genome sequencing (complete and partial) available at NCBI Genome BLAST, study of *Rickettsia conorii* subspecies *indica* infection confirmed by next-generation sequencing, Shandong, China

SRA read ID	Read length (nt)	% identity	Gene (chromosome location)	Rickettsia specificity
tRNA associated			(tRNA coverage/total)*	
SRR10855057.3634361.1	233	99.15	tRNA-Ala-GGC (45/76)	ITTR>R. conorii (Caspia, ISTT, Malish), other Rickettsia
SRR10855057.1770868.1	150	98.00	tRNA-Arg-TCT (77/77)	ITTR = Malish = Tenjiku01 = Rickettsia endosymb Proechinophthirus>other Rickettsia
SRR10855057.3222332.1	182	100.00	tRNA-Asn-GTT (5′ flank)	Many Rickettsiae, R. conorii (Caspia, ITTR, Malish) >R. conorii (ISTT)
SRR10855057.2512399.1	249	99.60	tRNA-Cys-GCA (75/75)	ITTR = R. parkeri, R. sibirica mong.>R. conorii (3), other Rickettsia
SRR10855057.2413080.1	184	98.92	tRNA-Gln-TTG (15/74)	ITTR = Malish>R. conorii (Caspia, ISTT), other Rickettsia
SRR10855057.3506905.1	240	98.17	tRNA-Glu-TTC (52/75)	ITTR = Malish>R. conorii (Caspia, ISTT), other Rickettsia
SRR10855057.2889951.1	239	98.76	tRNA-Glu-TTC (75/75)	ITTR = Malish>R. conorii (Caspia, ISTT), other Rickettsia
SRR10855057.882327.1	228	100.00	tRNA-His-GTG (77/77)	ITTR = Malish>R. conorii (ISTT, Caspia), other Rickettsia
SRR10855057.3412805.1	115	97.41	tRNA-His-GTG (14/77)	ITTR = Malish other Rickettsia>R. conorii (Caspia, ISTT)
SRR10855057.819370.1	150	99.34	tRNA-His-GTG (11/77)	ITTR = Malish = other Rickettsia>R. conorii (Caspia, ISTT)
SRR10855057.1459281.1	226	98.66	tRNA-Ile-GAT (76/77)	ITTR>R. conorii (Malish, ISTT, Caspia), other Rickettsia
SRR10855057.2012331.1	248	100.00	tRNA-Leu-GAG (5′ flank)	ITTR = Malish = Caspia>R. conorii ISTT, other Rickettsia
SRR10855057.1580324.1	216	97.72	tRNA-Leu-TAA (86/86)	ITTR = Malish = R. parkeri = R. sibirica mong.>R. conorii (ISTT, Caspia), other Rickettsia
SRR10855057.3515495.1	243	99.59	tRNA-Leu-TAA (80/86)	ITTR = Malish>R. conorii (ISTT, Caspia), other Rickettsia
SRR10855057.1645045.1	240	99.17	tRNA-Leu-TAA (3′ flank)	ITTR>R. conorii (Malish, ISTT, Caspia), other Rickettsia
SRR10855057.2952780.1	160	100.00	tRNA-Leu-TAA (3′ flank)	ITTR>Malish = R. parkeri = R. slovaca
SRR10855057.529019.1	227	98.68	tRNA-Lys-TTT (76/76)	ITTR>R. conorii (Malish, Caspia, ISTT), other Rickettsia
SRR10855057.796844.1	264	100.00	tRNA-Phe-GAA (3′ flank)	R. conorii (Malish, Caspia, ITTR)>R. conorii ISTT, other Rickettsia
SRR10855057.2538618.1	108	99.07	tRNA-Phe-GAA (24/76)	R. conorii (Malish, Caspia, ITTR), R. parkeri, R.sibirica>R. conorii ISTT, other Rickettsia
SRR10855057.2148777.1	208	99.04	tRNA-Pro-TGG (5′ flank)	ITTR = Malish>R. conorii (ISTT, Caspia), other Rickettsia
SRR10855057.1901026.1	210	99.52	tRNA-Pro-TGG (5′ flank)	ITTR = Malish>R. conorii (ISTT, Caspia), other Rickettsia
SRR10855057.2442098.1	119	93.39	tRNA-Pro-TGG (8/77)	Many Rickettsia
SRR10855057.3656176.1	135	96.92	tRNA-Pro-TGG (62/77)	Many Rickettsia
SRR10855057.2888707.1	98	96.88	tRNA-Pro-TGG (73/77)	Many Rickettsia
SRR10855057.650629.1	254	98.45	tRNA-Ser-GCT (3′ flank) (h prot)	ITTR = R. slovaca, R. africae>R. conorii (Malish, Caspia, ISTT) other Rickettsia
SRR10855057.108521.1	156	100.00	tRNA-Ser-GGA (37/88)	ITTR>R. conorii (Malish, Caspia, ISTT), other Rickettsia
SRR10855057.1718882.1	176	100.00	tRNA-Ser-GGA (88/88)	ITTR>R. conorii (Malish, Caspia, ISTT), other Rickettsia
SRR10855057.1545323.1	241	98.77	tRNA-Ser-TGA (89/90)	ITTR = Malish>R. conorii (Caspia, ISTT), other Rickettsia
SRR10855057.2148249.1	222	100.00	tRNA-Thr-CGT (5′ flank) (bamA)	ITTR>R. conorii (Malish, Caspia, ISTT), other Rickettsia
SRR10855057.3442561.1	223	99.55	tRNA-Thr-CGT (5′ flank) (bamA)	ITTR>R. conorii (Malish, Caspia, ISTT), other Rickettsia
SRR10855057.850105.1	221	98.66	tRNA-Thr-TGT (10/75)	ITTR = Malish, R. peacockii>other Rickettsia
SRR10855057.3110358.1	248	97.64	tRNA-Val-GAC (71/77)	ITTR = Malish>other Rickettsia, R. conorii (ISTT, Caspia)
RNA subunit (M1 RNA) of ribonuclease P, rnpB				
SRR10855057.2420743.1	164	100.00	rnpB	ITTR = Malish>R. conorii (Caspia, ISTT), other Rickettsia
16S ribosomal RNA			16S rRNA (rrs)	
SRR10855057.206203.1	226	100.00	rrs	Many Rickettsia including R. conorii (Malish, ITTR, Caspia, ISTT)
SRR10855057.991444.1	212	99.53	rrs	Many Rickettsia including R. conorii (Malish, ITTR, Caspia, ISTT)
SRR10855057.1633641.1	150	96.23	rrs	R. canadensis>other Rickettsia
SRR10855057.1948617.1	106	96.23	rrs	Bemisia Rickettsia, R. bellii>other Rickettsia
SRR10855057.2305806.1	180	99.42	rrs	Many Rickettsia including R. conorii (Malish, ITTR, Caspia, ISTT)
SRR10855057.3297795.1	210	99.30	rrs	Many Rickettsia including R. conorii (Malish, ITTR, Caspia, ISTT)

SRA read ID	Read length (nt)	% identity	Gene (chromosome location)	Rickettsia specificity
SRR10855057.3401137.1	200	99.50	rrs	Many Rickettsia including R. conorii (Malish, ITTR, Caspia, ISTT)
23S ribosomal RNA			23S rRNA (rrl)	
No reads mapping				No matches found
Citrate synthase			gltA	
SRR10855057.1205776.1	228	100.00	gltA	ITTR = Malish>R. conorii (Caspia, ISTT), other Rickettsia
SRR10855057.2326332.1	182	100.00	gltA	R. conorii (ITTR = Malish = Caspia = ISTT), other Rickettsia
SRR10855057.3271726.1	229	100.00	gltA&3′ flank	ITTR = Malish>R. conorii (Caspia, ISTT), other Rickettsia
Outer membrane protein OmpB			ompB (sca5)	
SRR10855057.3323415.1	204	97.58	ompB (sca5) and 5′ flank	ITTR>R. conorii (Caspia, ISTT, Malish), other Rickettsia
SRR10855057.1268928.1	231	100.00	ompB (sca5)	ITTR = Malish>other Rickettsia
SRR10855057.436084.1	213	96.36	ompB (sca5)	ITTR = Malish>other Rickettsia
SRR10855057.1634014.1	222	98.66	ompB (sca5)	ITTR>R. conorii (Caspia, ISTT, Malish), other Rickettsia
SRR10855057.2155308.1	155	100.00	ompB (sca5)	ITTR = Malish>other Rickettsia
SRR10855057.2143700.1	223	100.00	ompB (sca5)	ITTR = Malish>other Rickettsia
SRR10855057.3503391.1	217	99.54	ompB (sca5)	ITTR>R. conorii (Caspia, ISTT, Malish), other Rickettsia
Outer membrane protein OmpA			ompA (sca0)	
SRR10855057.1793457.1	232	100.00	ompA (sca0) and 5′flank	ITTR>R. conorii (Malish, Caspia, ISTT), other Rickettsia
SRR10855057.1750933.1	232	95.34	ompA (sca0)	ITTR = Malish>R. conorii (Caspia, ISTT), other Rickettsia
SRR10855057.1660860.1	147	95.24	ompA (sca0)	R. conorii (ITTR = Malish = ISTT = Caspia) >other Rickettsia
SRR10855057.810562.1	262	100.00	ompA (sca0)	ITTR = Malish>R. conorii (Caspia, ISTT), other Rickettsia
SRR10855057.790884.1	245	100.00	ompA (sca0)	ITTR = Malish>R. conorii (Caspia, ISTT), other Rickettsia
SRR10855057.611967.1	200	99.50	ompA (sca0)	ITTR = Malish>R. conorii (Caspia, ISTT), other Rickettsia
SRR10855057.249559.1	200	94.76	ompA (sca0)	R. conorii (Caspia, Malish, ITTR, ISTT) = R. africae = R. parkeri = R. sibirica>other Rickettsia
Cell surface antigen 4			sca4	
No reads mapping				
Pseudogene RC_RS06985-IGS				
SRR10855057.210346.1	233	99.57	RC_RS06985 (3′ flank)	ITTR>R. conorii (Malish, ISTT, Caspia), other Rickettsia
SRR10855057.1977290.1	183	99.46	RC_RS06985 (3′ flank)	ITTR>R. conorii (Malish, ISTT, Caspia), other Rickettsia
SRR10855057.3088753.1	183	98.92	RC_RS06985 (3′ flank)	ITTR>R. conorii (Malish, ISTT, Caspia), other Rickettsia
SRR10855057.3018756.1	176	100.00	RC_RS06985 (3′ flank)	ITTR>R. conorii (Malish, ISTT, Caspia), other Rickettsia
UDP-3-O-(3-hydroxymyristoyl)glucosamine N-acyltransferase (luxD) RC_RS00050 (5′ flank)				
SRR10855057.3390182.1	210	100.00	lpxD (5′ flank)	ITTR = Malish>R. conorii (ISTT, Caspia), other Rickettsia
IGS of AsmA family protein RC_RS02405 and rimM RC_RS02410				
SRR10855057.2154203.1	242	99.59	asmA-rimM IGS	ITTR = Malish>R. conorii (ISTT, Caspia), other Rickettsia
Preprotein translocase subunit SecG RC_RS00570 (5′ flank) (tRNA-Thr-GGT)(3‘ flank)				
SRR10855057.65175.1	150	96.67	secG (5′ flank)	ITTR = Malish = R. parkeri, R. sibirica = R. slovaca>other Rickettsi
Rod shape-determining protein MreC RC_RS05910 (3′ flank)				
SRR10855057.870441.1	151	95.36	mreC (3′ flank)	ITTR>R. conorii (Malish, Caspia, ISTT), other Rickettsia

*Where indicated, numbers in brackets correspond to the total nucleotide length of the tRNA gene (denominator) and the number of tRNA nucleotides overlapping with a listed next-generation sequencing read (numerator). IGS = intergenic spacer. Reads providing specific identification of *Rickettsia conorii* subspecies *indica* infection are shown in gray. ITTR, *R. conorii indica*; Caspia, *R. conorii caspia*; ISTT, *R. conorii israelensis*; Malish, *R. conorii conorii* strain Malish.

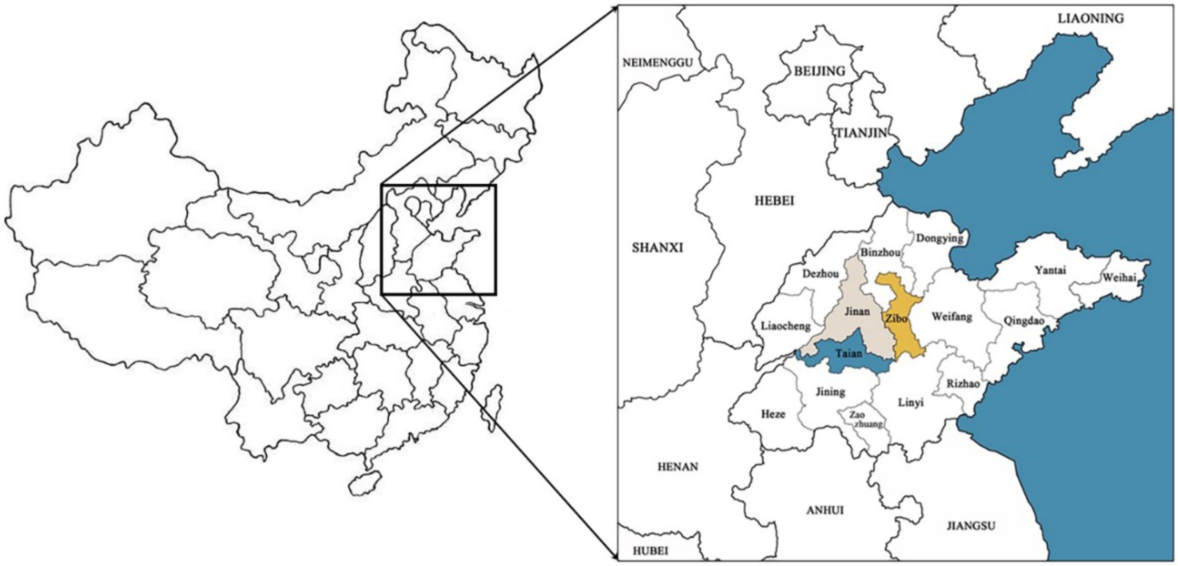

Appendix Figure 1. Map of the geographic locations where exposure to rickettsial pathogens occurred, Shandong Province, China.

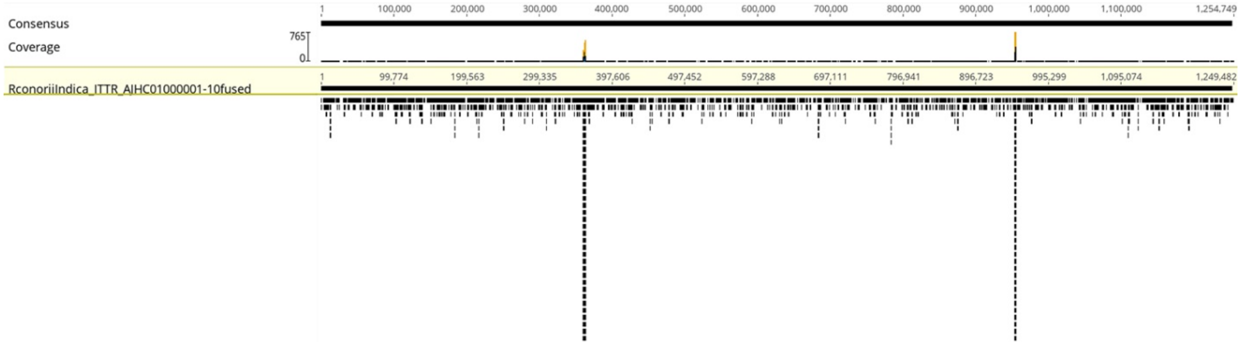

Appendix Figure 2. Mapping by Geneious Prime BLAST of 9581 Ion Torrent reads to the *Rickettsia conorii indica* genome (AJHC01000001.1–10.1) including 10 fused contigs which are syntenic to the complete *R. conorii conorii* Malish 7 genome (NC_003103.1). The large pileups of 16S rRNA (coordinates 359040–361800; 3,991 reads) and 23S rRNA (coordinates 950294–951801; 2,641 reads) gene reads are truncated in the figure.
